# Use of evidence-based health professions education: Attitudes and practices of academic leaders

**DOI:** 10.1371/journal.pone.0314772

**Published:** 2025-01-28

**Authors:** Shazia Nawabi, Rida Fatima Waseem, AbdulAziz Binrayes, Ayman Moaz Abulhamael, Zaina Ahmad, Kiran Imtiaz Khan, Syed Rashid Habib, Muhammad Qasim Javed

**Affiliations:** 1 Department of Prosthodontics, HBS Medical and Dental College, Islamabad, Pakistan; 2 Department of Operative Dentistry and Endodontics, Islamabad Medical and Dental College, Islamabad, Pakistan; 3 Department of Prosthetic Dental Sciences, College of Dentistry, King Saud University, Riyadh, Kingdom of Saudi Arabia; 4 Faculty of Dentistry, Department of Endodontics, King Abdulaziz University, Jeddah, Saudi Arabia; 5 House Officer, Islamic International Dental College and Hospital, Riphah International University, Islamabad, Pakistan; 6 Pediatric Dentistry, Army Medical College, National University of Medical Sciences, Rawalpindi, Pakistan; 7 Department of Conservative Dental Sciences, College of Dentistry, Qassim University, Buraidah, Qassim, Saudi Arabia; Thamar University: Dhamar University, YEMEN

## Abstract

**Purpose:**

The objective of this study was to explore the attitudes, practices, supports, and barriers of academic leaders regarding the use of Evidence-Based Health Professional Education (EBHPE).

**Methods:**

A cross-sectional survey was conducted on 79 faculty members in leadership positions, from four different undergraduate colleges at Qassim University. A pre-validated questionnaire was distributed electronically. The e-questionnaire was comprised of 38 questions regarding participants’ demographics, attitudes, practices, and perceived barriers/supports towards EBHPE. Data was analyzed using SPSS. Descriptive statistics were calculated for demographic characteristics and responses to survey items. The frequency distribution of the subjects was analyzed, and the median and means were calculated. Kruskall-Wallis and Mann-Whitney U tests were used for comparison of the means between the demographic groups. Spearman correlation was utilized to determine any relationships between the questionnaire’s three sections.

**Results:**

Of the 79 participants, 24 were Department Heads and 48 were female. The mean±SD values for Attitude, Practices and Support/Barriers were found to be 3.47±0.40, 2.72±0.84 and 3.34±0.43, respectively. The mean value of Attitude Score was > 3.41, therefore, it was interpreted that respondents had positive attitude towards EBHPE. Conversely, the values for Practices and Support/Barriers were interpreted as neutral (range = 2.61–3.40), neither good nor bad. The correlation coefficients between ’attitudes’ and ’supports and barriers’ (0.388) and ‘practices’ and ‘supports and barriers’ (0.388) indicated the weak association between these factors. The Cronbach’s α value for questionnaire was found to be 0.887 indicating its reliability.

**Conclusions:**

This study concludes that the respondents had positive attitude towards EBHPE. However, this positive attitude was not reflected in their practices. Healthcare professionals should be committed to educational excellence, and endeavour to rely on evidence-based literature regarding the planning and review of learning, teaching, and assessment strategies.

## Introduction

The complex and rapidly transforming healthcare system demands healthcare professionals to continuously upgrade their knowledge and skills to meet intricate patient and healthcare challenges. Training health professionals to acquire aforementioned competencies is likewise complex [1]. These contemporary complexities of health professions education necessitate academic leaders to adapt innovative and scientifically proven educational strategies used in a variety of diverse venues [2]. Therefore, the method and approach based on the best evidence available hold an important place alongside the teachers’ traditional comfort zone.

Evidence-based practice is an approach to decision-making that has permeated all aspects of healthcare. While the concept originated in medicine, it has influenced a wide range of health professions [3]. Health professions education has also moved on from the era of judgment-based education to evidence-based education. The concept of evidence-based health professions education (EBHPE) was advocated by the world’s eminent leaders in the field of medical education, R.M. Harden and Jannet Grant, more than two decades ago in BEME guide 1 [4]. The Best Evidence Medical Education Collaboration (BEME) is an international group of individuals, universities and professional organizations founded by R.M. Harden in 1999. The collaboration is committed to the development of evidence-informed education by supporting researchers in health professions education and making available the latest findings from scientifically grounded educational research in the series of BEME guides [5]. Guides have been further analysed to estimate their readiness for integration into practice [6].

However, the application of evidence into practice requires a strong organizational infrastructure, committed to supporting the delivery of evidence-based health education along with encouraging healthcare professionals to acquire EBHPE competencies [7]. This emphasizes the heightened need for effective academic leadership One study by Alimo-Metcalfe B and Alban-Metcalfe RJ highlighted the three ‘top qualities’ required of academic leaders from a ‘follower’ perspective. These were: having a genuine concern for others, being inspirational communicators, and having the ability to empower others [8]. Successful educational leadership controls the institutional balance between the applicability of feasible evidence-informed educational reform and available infrastructure.

Academic leadership is different from corporate leadership exercised in business management, which indicates that leadership is a context-based phenomenon that varies across situations [9]. Bennis in 1989 suggested that leadership is like beauty: hard to define but one can appreciate it once around [10]. To address this complexity, a lot of literature was published attempting to capture the essence of leadership in the epoch of the early 21^st^ century. However, the evolution of interwoven leadership ideologies complicates the picture with no clear consensus on the definition of leadership.

Academic leaders, at all levels, have a significant impact on the development and performance of an educational organization. Therefore, effective leadership is an essential feature of administrative and managerial positions in medical colleges and universities [11] Top administration, heads of departments, and course directors have an exclusive role as academic leaders. They are often called upon to apply an evidence-informed approach to educational reforms as members of curriculum teams and assessment projects. There is a lot of recent literature on attitudes and practices regarding evidence-based clinical practice [12–14], but there is limited rational research investigating academicians’ attitudes and practices towards the use of research evidence in health education practice.

The present study was designed to explore the perceptions and practices of academic leaders in administrative positions including deans, vice deans, course directors, heads of departments, and medical educationists regarding evidence-informed health professions education (HPE). Their perceived barriers and supports regarding the matter were also assessed. The study was further designed to observe/compare the hierarchal pattern of beliefs in the application of evidence-informed educational practices among administrative authorities.

## Materials and methods

The study consisted of a cross-sectional survey of faculty of Qassim University College of Medicine, dentistry, nursing, and physiotherapy/pharmacy at different leadership positions, i.e. head of department, block organizer, chairpersons, vice deans, deans, and program directors. The sample size (SS) of 79 was calculated as acceptable SS by using qualtrics SS calculator, with 90% confidence level, population size (faculty in leadership position) of 110, and 5% margin of error [15]. The study was granted ethical approval from the Qassim University review board (Approval no: ST/6073/2020, dated: 18/07/2020). The study was conducted from 1^st^ August to 31^st^ October 2020. The participation of all the respondents was voluntary.

After obtaining permission from Dr. Aliki Thomas, the study questionnaire was adopted from pre-validated survey instrument developed by Dr Aliki Thomas and colleagues [16]. The original questionnaire consisted of 49 items distributed across 4 sections. A team of five senior health profession educators and psychometrician were involved to review the original instrument as per our context and objective of the study. The quantitative assessment for content validity index [CVI]; content validity; and content validity ratio [CVR] was carried out for the thirty questionnaire items in section 2,3, and 4. The CVR was ascertained by requesting the team members to score each item utilizing three point likert scale (LS) [‘necessary’/ ‘beneficial but not necessary’/ ‘not necessary’]. The CVI was determined by the same team using four point LS on ‘simplicity’/ ‘relevancy’/ ‘clarity’. The calculation of CVI was done by adding each item’s positive scores divided by total team members. The 0.7 was set as the cut off value for retaining the items. The final questionnaire’s face validity was ensured by assessing the clarity and grammar. Subsequently, the questionnaire was pilot tested with a subset of educators (n = 10) representative of our target population and they were requested to evaluate each item for difficulty and vagueness, while replying to the questionnaire. The data from pilot study was not included in final data analysis. Final questionnaire consisted of 38 items divided across four sections. Section 1 was comprised of 8 questions related to participants’ demographics, including their organization, designation, leadership position, age, gender, qualification (especially in HPE) and years of experience. The questionnaire in sections 2,3 and 4, was comprised of 30-items divided into three sections, each having 10 items regarding attitudes, practices, and perceived barriers. The questionnaire responses for attitudes and supports/barriers were evaluated using a five-point Likert scale (LS), with ’1’ indicating Strongly Disagree and ’5’ indicating Strongly Agree. Negative statements were reverse scored. Likewise, practices were scored on a five-point LS with ’1’ indicating Never, ’2’ indicating Rarely, ’3’ indicating Sometimes, ’4’ indicating Often, and ’5’ indicating Very Often. The scores were then aggregated under each of the three categories i.e. attitudes, practices and supports/ barriers and the total score was also calculated for each participant. Higher scores reflected a more positive attitude towards evidence-based medical education, better practices of evidence-based medical education, or greater opportunities provided. To interpret the scores, class intervals outlined by Doronilla were used [17].

The participation information sheet, consent form and study questionnaire was distributed electronically to the target population. Subsequently, two reminders were given at 3 weeks’ interval. The research objective was explained to potential participants via information sheet. An informed consent was obtained in written form electronically.

### Statistical analysis

Data was analyzed using SPSS v26. Descriptive statistics were carried out to describe both the demographic characteristics as well as the responses for each survey item corresponding to attitudes, practices, and perceived barriers. The frequency distribution of the subjects was analyzed, and the median and means were calculated. The five point LS responses for sections 2,3, and 4, were converted to three categories. The responses for attitude (Section 2) and Barriers/Supports (Section 4); Disagree {DA}, and Strongly disagree {SDA} were collapsed to “DA”, and Strongly Agreed {SA} was merged with Agreed {A} to form “Agree”. On the other hand, responses for practices (Section 3); Often {O}, and Very Often {VO} were combined to “often”, and Never {N} and Rarely {R} formed “Never/Rarely”. The Shapiro-Wilk test indicated a mixed data set in terms of normality. As a result, non-parametric tests (Kruskall-Wallis and Mann-Whitney U tests) were used for comparison of the means between the demographic groups. Spearman correlation was utilized to determine any relationships between the questionnaire’s three sections i.e. attitudes, practices and supports/barriers. Additionally, the percentage of responses for each item was calculated. The reliability of the questionnaire assessing educators’ attitudes, practices, perceived barriers, and supports was evaluated using two reliability measures: Cronbach’s α and McDonald’s ω. The reliability of the model was assessed using the chi-square test and the Kaiser-Meyer-Olkin (KMO) measure. A Factor Analysis was conducted to identify the underlying factors. Model fit was determined by RAMSEA, TLI and BIC.

## Results

A total of 79 individuals completed the questionnaire, with the majority being female (61%). Among the respondents, 46.8% fell into the 40–50 age bracket, 30.4% held positions as Heads of Departments, and most possess a Master/MPhil in Medical Education. Table 1 provides a detailed breakdown of the demographics, presenting the mean scores across different groups and their respective p-values.

**Table 1:** Association between demographic variables and attitude, practice, support, and barriers mean scores.

Demographic	Practices Median (IQR)	Practices p value	Attitudes Median (IQR)	Attitudes p value	Supports and barriers Median (IQR)	Supports and barriers p value	Overall Sum Median (IQR)	p-value*
**Gender**								
Male (n = 31)	3.00 (3.00)	0.181	3.50 (1.30)	0.306	3.40 (2.10)	0.087	10.00 (5.90)	0.122
Female (n = 48)	2.70 (3.90)		3.50 (2.90)		2.70 (3.90)		9.50 (5.30)	
**Age Group**								
25–30 (n = 7)	2.60 (2.10)	0.766	3.20 (0.90)	0.277	3.10 (0.80)	0.159	8.70 (3.30)	0.285
30–40 (n = 27)	2.50 (2.90)		3.60 (2.00)		3.30 (2.10)		9.50 (6.00)	
40–50 (n = 37)	2.90 (4.00)		3.50 (2.20)		3.30 (1.70)		9.90 (5.20)	
50–60 (n = 8)	3.00 (2.40)		3.60 (0.90)		3.35 (0.70)		10.05 (3.40)	
**Academic Position**								
Vice Dean (n = 2)	2.15 (2.10)	0.180	3.35 (0.90)	0.306	3.05 (0.10)	0.386	8.55 (2.90)	0.387
Course Coordinator (n = 9)	2.10 (2.20)		3.60 (1.20)		3.30 (1.50)		8.80 (4.00)	
Head of Department (n = 24)	2.80 (2.90)		3.40 (2.10)		3.20 (1.40)		9.40 (5.00)	
Chair Academic Committee (n = 10)	3.15 (2.30)		3.70 (1.00)		3.30 (1.20)		10.15 (3.20)	
Block Organizer(n = 9)	3.00 (2.00)		3.60 (0.70)		3.60 (1.30)		10.00 (2.90)	
Medical/Dental Educationist (n = 5)	2.90 (2.90)		3.20 (0.70)		3.40 (0.90)		9.50 (2.00)	
Teacher (n = 17)	2.40 (2.70)		3.50 (2.10)		3.30 (2.10)		9.30 (5.90)	
Other (n = 3)	3.30 (2.20)		3.40 (2.20)		3.20 (0.50)		10.10 (2.20)	
**Medical Education Qualification**								
PhD (n = 10)	3.30 (3.10)	0.273	3.50 (1.10)	0.745	3.25 (1.50)	0.371	10.25 (4.50)	0.802
Masters/Mphil (n = 23)	2.90 (4.00)		3.50 (1.30)		3.30 (1.70)		9.90 (5.00)	
Diploma (n = 5)	3.00 (2.30)		3.30 (1.00)		3.00 (0.40)		9.50 (2.90)	
Certificate (n = 2)	3.25 (1.10)		3.10 (0.60)		3.10 (0.20)		9.45 (1.50)	
Workshops/ Others (n = 17)	2.50 (2.20)		3.60 (2.10)		3.40 (1.60)		9.70 (5.30)	
None (n = 22)	2.50 (2.50)		3.50 (1.90)		3.30 (2.10)		9.30 (4.80)	
**Years of Practice**								
<5 (n = 7)	2.30 (1.90)	0.543	3.40 (0.80)	0.631	3.30 (1.90)	0.644	8.80 (4.20)	0.663
6–10 (n = 21)	2.70 (2.60)		3.60 (2.90)		3.20 (1.60)		9.40 (5.30)	
11–15 (n = 24)	2.85 (3.20)		3.50 (1.30)		3.30 (1.30)		10.00 (4.80)	
16–20 (n = 16)	2.85 (3.10)		3.50 (1.20)		3.30 (1.50)		9.50 (4.00)	
21–25 (n = 7)	3.00 (2.60)		34.00 (0.70)		3.40 (0.80)		10.00 (3.40)	
>26 (n = 4)	3.05 (2.50)		3.80 (1.20)		3.30 (0.90)		10.30 (4.10)	

*Kruskal Wallis test and Mann Whitney u test were used to obtain p values. p<0.05 significant.

No significant differences (p<0.05) were detected among the demographic groups.

Table 2 displays the breakdown of respondents’ attitudes toward the utilization of evidence in health professions education. Most respondents were open to changing their teaching methodologies based on the latest research finding. A significant portion of participants believed that evidence-informed approach to education improves the quality of educational practices. Additionally, most respondents remained neutral about the importance of student course evaluations being more important than medical education research findings in guiding the decisions. Only 26.6% of respondents disagreed that Medical education is heavily guided by intuitions.

**Table 2:** Percentage of responses to items concerning attitudes towards evidence based health professions education.

	Statement (Attitudes)	% (freq) Strongly Disagree/ Disagree	% (freq) Neutral	% (freq) Strongly Agree/ Agree
**1**	“I draw from medical education research findings when I make important decisions about educational practices”	8.9 (7)	24.1 (19)	67.1 (53)
*2	“I would rather learn about educational practices from colleagues than from the scientific literature”	64.6 (51)	20.3 (16)	15.2 (12)
**3**	“I am open to changing my teaching methods based on the latest research findings”	10.1 (8)	6.3 (5)	83.5 (66)
*4	“Data from student course evaluations are more important than medical education research findings in guiding the decisions I make about educational practices”	25.3 (20)	39.2 (31)	35.4 (28)
*5	“Educators in the medical professions make little use of educational research findings”	29.1 (23)	35.4 (28)	35.4 (28)
*6	“An evidence-informed approach to education is too time-consuming”	40.5 (32)	27.8 (22)	31.6 (25)
**7**	“An evidence-informed approach to education improves the quality of educational practices”	11.4 (9)	11.4 (9)	77.2 (61)
**8**	“If we take research evidence seriously, our educational practices will look quite different to the way they do now”	13.9 (11)	13.9 (11)	72.2 (57)
**9**	“Medical education research findings are applicable to educational practice”	8.9 (7)	17.7 (14)	73.4 (58)
*10	“Medical education is heavily guided by intuitions”	26.6 (21)	36.7 (29)	36.7 (29)

The practices of the participants regarding the use of evidence in health professions education are presented in Table 3. A total of 62% of the respondents stated frequent attendance at faculty development or professional development activities within the past year. In contrast, a mere 8.9% reported frequent presentations at medical education conferences over the same period. Likewise, only 11.4% indicated regular publication of papers related to medical education.

**Table 3:** Percentage of responses to items concerning practices in evidence-based health professions education.

	Statement (Practices) In the last year how often you have?	% (freq) Never/ rarely	% (freq) Sometimes	% (freq) Often/ Very Often
**1**	“Attended conferences or scientific meetings on medical education”	41.8 (33)	36.7 (29)	21.5 (17)
**2**	“Attended faculty development/continuing professional development activities”	19.0 (15)	19.0 (15)	62.0 (49)
**3**	“Engaged in curriculum design and development/ program evaluation	39.2 (31)	32.9 (26)	27.8 (22)
**4**	“Engaged in medical education research”	44.3 (35)	29.1 (23)	26.6 (21)
**5**	“Searched for scientific articles in education”	31.6 (25)	27.8 (22)	40.5 (32)
**6**	“Written scholarly papers in medical education”	59.5 (47)	29.1 (23)	11.4 (9)
**7**	“Presented at a medical education conference”	77.2 (61)	13.9 (11)	8.9 (7)
**8**	“Used medical education research findings to inform teaching in the classroom/clinical setting”	36.7 (29)	35.4 (28)	30.4 (22)
**9**	“Used medical education research findings to assess learners”	34.2 (27)	35.4 (28)	30.4 (24)
**10**	“Used medical education research findings to inform administrative educational decisions”	41.8 (33)	29.1 (23)	29.1 (23)

Table 4 displays the items related to the opportunities and obstacles influencing the adoption of evidence-based practice in medical education. 70.9% of the participants were confident in their ability to be an evidence-informed educator. A substantial number of respondents acknowledged the utility of faculty development activities in applying research findings to educational methods. 62% of the participants reported the availability of faculty development opportunities offered by their organization. However, 38% of the respondents perceived a reluctance among teachers within their organization to embrace changes in educational practices.

**Table 4:** Percentage of responses to items concerning supports and barriers in evidence-based health professions education.

	Statement (supports and barriers)	% (freq) Strongly Disagree/ Disagree	% (freq) Neutral	% (freq) Strongly Agree/ Agree
**1**	“I have sufficient time to read the literature on medical education”	24.1 (19)	40.5 (32)	35.4 (28)
**2**	“I have access to current medical education research (electronic or paper)”	15.2 (12)	25.3 (20)	59.5 (47)
*3	“I have difficulty judging the medical education literature”	40.5 (32)	45.6 (36)	13.9 (11)
*4	“I cannot keep up with the volume of medical education research”	25.3 (20)	43.0 (34)	31.6 (25)
**5**	“My organization gives me opportunities for faculty development/continuing professional development”	15.2 (12)	22.8 (18)	62.0 (49)
*6	“Teachers in my organization are reluctant to change educational practices”	38.0 (30)	25.3 (20)	36.7 (29)
*7	“Leadership in my organization is reluctant to change educational practices”	49.4 (39)	26.6 (21)	24.1 (19)
**8**	“Faculty development/continuing professional development activities help me apply research findings to educational practices”	12.7 (10)	21.5 (17)	65.8 (52)
**9**	“New medical education research findings are regularly disseminated in my work environment”	19.0 (15)	36.7 (29)	44.3 (35)
**10**	“I am confident in my ability to be an evidence-informed educator”	11.4 (9)	17.7 (14)	70.9 (56)

Among the three categories, respondents scored the highest in their ‘attitude’ toward utilizing evidence in health professions education, with a mean of 34.73 (SD = 4.03) and a median of 35.00. This indicates that participants had the most positive attitude towards evidence-based medical education. However, the ‘practice’ category had the lowest scores, with a mean of 27.20 (SD = 8.36) and a median of 28.00.

The mean ± Standard deviation values for Attitude, Practices and Support/Barriers were found to be 3.47±0.40, 2.72±0.84 and 3.34±0.43, respectively. The median values for Attitude, Practices and Support/Barriers were found to be 3.50, 2.80 and 3.30, respectively. Whereas, interquartile range (IQR) for Attitude, Practices and Support/Barriers was found to be 2.90, 4.00 and 2.60, respectively.

Table 5 presents the interpretative values used in our study, as outlined by Doronilla et al. [17]. According to this interpretation, the mean and median values for attitudes fall within the "agree" category, while the mean and median values for practices indicate that the data primarily falls within the "neutral" category.

**Table 5:** Interpretative values for attitudes and practices based on Doronilla [17].

Mean Score	Interpretation	Attitude/Practices score interpretation
**4.21–5.00**	Strongly Agree	Attitude Score value ≥ 3.41 is considered as positive. Practices score ≥ 3.41 is considered as good.
**3.41–4.20**	Agree	
**2.61–3.40**	Neutral	Neutral attitude and Practice
**1.81–2.60**	Disagree	Attitude Score value ≤ 2.60 is considered as negative. Practices score ≤ 2.60 is considered as poor.
**1.00–1.80**	Strongly Disagree	

The correlation coefficients between ’attitudes’ and ’supports and barriers’ (0.388) and ‘practices’ and ‘supports and barriers’ (0.388) indicated the weak association between these factors though the p-values indicated significant results (p<0.05).

CVR and CVI for all 30 items were acceptable (ranging from 0.81 to 0.93). The reliability of the scale assessing educators’ attitudes, practices, perceived barriers, and supports was examined using Cronbach’s α (0.887) and McDonald’s ω (0.909), both of which indicate excellent internal consistency, confirming that the instrument reliably measures its intended constructs.

The reliability of the model was assessed using the chi-square test and the Kaiser-Meyer-Olkin (KMO) measure. The chi-square value (χ^2^ = 1558, df = 435, p < 0.001) indicates that there is a significant difference between the observed and expected data, suggesting that the model does not perfectly fit the data. However, given that the chi-square test is sensitive to large sample sizes, this result does not necessarily imply a poor model fit. The KMO measure of sampling adequacy was 0.780, indicating that the sample is adequate for factor analysis. A KMO value between 0.7 and 0.8 is considered acceptable, demonstrating that there is sufficient common variance in the data for factor analysis to be appropriate. Overall, while the chi-square test points to areas for potential model improvement, the KMO value supported the data’s suitability for further analysis.

Factor analysis revealed four distinct factors: Factor 1: Research-Informed Teaching Practices, Factor 2: Access and Resources for Educational Research, Factor 3: Challenges in Engaging with Research and Factor 4: Organizational and Leadership Support for Educational Innovation (Table 6).

**Table 6:** Scale statement with factor loadings.

		Factors				
Sr#	Perceptions / Attitudes	1	2	3	4	Uniqueness
1	I draw from medical education research findings when I make important decisions about educational practices		0.754			0.399
2	I would rather learn about educational practices from colleagues than from the scientific literature	0.364			0.429	0.637
3	I am open to changing my teaching methods based on the latest research findings		0.900			0.250
4	Data from student course evaluations are more important than medical education research findings in guiding the decisions I make about educational practices		0.411	0.330		0.662
5	Educators in the medical professions make little use of educational research findings		0.514	0.354		0.529
6	An evidence-informed approach to education is too time-consuming		0.398			0.659
7	An evidence-informed approach to education improves the quality of educational practices		0.847			0.206
8	If we take research evidence seriously, our educational practices will look quite different to the way they do now		0.794			0.304
9	Medical education research findings are applicable to educational practice		0.808			0.381
10	Medical education is heavily guided by intuitions			0.315		0.737
	**Practices**					
1	Attended conferences or scientific meetings on medical education	0.758				0.424
2	Attended faculty development/continuing professional development activities		0.563			0.502
3	Engaged in curriculum design and development/ program evaluation	0.548				0.541
4	Engaged in medical education research	0.755				0.383
5	Searched for scientific articles in education	0.675				0.450
6	Written scholarly papers in medical education	0.798				0.388
7	Presented at a medical education conference	0.762				0.413
8	Used medical education research findings to inform teaching in the classroom/clinical setting	0.792				0.336
9	Used medical education research findings to assess learners	0.765				0.383
10	Used medical education research findings to inform administrative educational decisions	0.685				0.390
	**supports/barriers**					
1	I have sufficient time to read the literature on medical education					0.734
2	I have access to current medical education research (electronic or paper)	0.333			0.499	0.483
3	I have difficulty judging the medical education literature			0.546		0.620
4	I cannot keep up with the volume of medical education research			0.660		0.382
5	My organization gives me opportunities for faculty development/continuing professional development				0.552	0.479
6	Teachers in my organization are reluctant to change educational practices			0.824		0.345
7	Leadership in my organization is reluctant to change educational practices			0.800		0.377
8	Faculty development/continuing professional development activities help me apply research findings to educational practices				0.611	0.338
9	New medical education research findings are regularly disseminated in my work environment				0.589	0.386
10	I am confident in my ability to be an evidence-informed educator		0.333		0.558	0.352

A RMSEA (Root Mean Square Error of Approximation) value of 0.0681 suggests a reasonable model fit, as RMSEA values between 0.05 and 0.08 indicate an acceptable model fit. Lower values indicate better fit, and values below 0.05 would indicate a good fit. The confidence interval provides a range in which the true RMSEA lies. Since the lower bound (0.0531) is close to 0.05 and the upper bound (0.0851) is below 0.1, this further supports an acceptable fit.

The RMSEA value and its confidence interval suggest a reasonable model fit. The TLI (0.844) indicates that there may be room for improvement in model fit. The significant chi-square result (<0.001) suggests that the model does not perfectly capture the data, but this could be influenced by sample size. The negative BIC value (-960) is encouraging, suggesting that the model may be preferred when compared to others. In conclusion, the model has an acceptable but not perfect fit, with room for improvement based on the TLI and chi-square test results.

## Discussion

This study was designed to assess the attitude, practice, supports, and barriers of academic leaders in administrative roles, regarding EBHPE. The subset included the faculty of Qassim University College of Medicine, Dentistry, Nursing, and Physiotherapy/Pharmacy working at different leadership positions for Qassim University who have a direct impact on the formation of health professions education policies. The faculty is routinely involved in the design and implementation of the curriculum for undergraduate and postgraduate programs. This also entails modifications in the running curriculum where review and upgradation of the teaching and assessment strategies are often required. According to the results, most of the participants have had some formal medical education training in addition to their discipline based professional qualification, with masters/MPhil being the most common professional qualification. This indicates a positive change in the mindset of the faculty who have been trained to think as educators. Hence, the overall response to a change in teaching practices based on EBHPE was encouraging.

The demographic results suggest a correlation between age and experience with good educational practices. Respondents practicing for 21–25 years displayed a higher score in terms of opportunities compared to those with 6–10 years of practice as evidently, they gathered more exposure by working at different institutes and multiple positions in the course of their career. A similar trend was observed with the age of the participants where participants aged 50–60 scored significantly higher in attitudes and opportunities when compared to their 25–30 years old counterparts. In the early parts of their career, educators are more likely to be consumed by the progress and pace of their own careers rather than inculcating research-based educational practices in their teaching. As a part of continued educational programs, educators attend seminars or conferences that make them more aware and inclined to incorporate EBHPE in their classrooms [18].

Among the three attributes, it was noted that the participants scored the highest in their attitude towards evidence in health professions education. A significant number of the group disagreed that in decision-making practices, medical education research findings are more important than data from student course evaluations. This finding is in agreement with a study performed by Thomas et al where members of the Association for Medical Education in Europe also seemed to rely more on student course evaluations [16]. However, it is recognized now that student evaluations do not always paint a true picture of the classroom. The evaluations are at times done more as a mindless chore than an honest evaluation [19]. So, it would be prudent for administrative leadership to rely more on medical research evidence for high-stakes policy decisions rather than on student evaluations. In this manner, the policies can be justified and critically analysed. Similarly, the majority of the participants agreed to rely on intuition when compared with research-based health education. Although intuition has always been a considerable factor in clinical teaching, the balance has shifted in favour of evidence-based medicine. Likewise, the emerging body of research in medical education provides support to educators to reduce bias and uncertainty on both sides of the classroom that comes with intuitive educational decision-making [20].

Interestingly, it was encouraging that the participants did not seem to think that educators in the medical professions make little use of educational research findings. However, an evidence-based approach was considered time-consuming by most. Most faculty members struggle with juggling multiple roles in one position and consequently educating themselves about evidence from the field of medical education goes down their list. Interestingly, the group remained neutral on the question of whether an evidence-informed approach to education improves the quality of educational practices. This could be due to the conflict in theory and practice in most institutions regarding EBHPE. The reasons for this require to be explored further with research. During the instructional design process throughout the year, the teachers should have evidence-based guidance on the development of effective teaching strategies. In a systematic review regarding the evidence for incorporating Internet Based Learning (IBL) in different courses, results showed that although sufficient evidence is present to support the use of IBL but it remains unclear when it should be used [21].

The practices of the faculty of Qassim University showed that although most participants had exposure to faculty development/continuing professional development activities, it did not translate into greater production of medical education literature. Similarly, although many participants had searched for scientific articles on medical education, the responses to the practical use of literature in informed teaching in classrooms, assessment, and educational policy-making were neutral. These findings could be due to a dearth of good quality research and a lack of understanding of clinical teachers of the jargon of medical education literature [22]^.^ At Qassim University a hybrid outcome-based integrated curriculum is in practice where teaching strategies such as lectures as well as Task Based Learning and Problem-Based Learning are being utilized. Additionally, basic sciences skills lab and clinical rotations are run for the psychomotor learning of their students. These strategies require extensive resources including the presence and time of the faculty for smooth execution. In addition to this, the faculty is also responsible for administrative roles such as course coordination, student evaluations, quality assurance, and record keeping. They are working as clinicians and mentors simultaneously. All these challenging roles are fulfilled by the faculty members at the same time making it difficult to continue being updated in medical education literature and its practical implications. One possible solution could be to include medical education research in mainstream medical journals to increase their reach [20]. Another solution could be that the faculty has designated time weekly or fortnightly to meet and discuss medical education literature with their peers. This could also lead to developing research collaborations amongst different disciplines at Qassim University.

When considering supports and barriers, there was a positive response regarding the availability of medical education literature. However, the participants encountered challenges in keeping up with the extensive volume of available research, which was acknowledged as a significant barrier in EBHPE. Kabirpanthi et al also identified extrinsic barriers such as workload and lack of time for research. Most participants agreed that there were ample opportunities for the faculty for continued professional development which helped them to apply research findings to educational practices in their organization. The faculty seemed positive regarding the willingness of management and faculty in terms of changing educational practices. This contrasts with previous studies by Thomas et al and Onyura et al, both of whom reported that the status quo was reluctant to bring change in educational practices based on evidence-based research. Onyura et al. reported four main barriers to research use including poor quality and availability of evidence, inadequate knowledge delivery approaches, work and role overload, and faculty and student resistance to change. However, in the population used in our study, these barriers were not recognized except for keeping up with the volume of available literature [23]. Due to these positive trends, a significant number were confident in their ability to be an evidence-informed educator.

This study revealed a weak association between attitudes towards EBHPE and the supports and barriers perceive. This suggests that while individuals might possess positive attitudes or beliefs regarding research-based education, various factors may hinder the translation of these attitudes into actual practices. Possible barriers could be resource limitations or challenges in integrating research-based methodologies into the educational curriculum or clinical settings. Similarly, the weak correlation between practices and opportunities might indicate that despite the existence of opportunities for research-based education, these opportunities might not be effectively embraced by individuals in their practices potentially due to a lack of awareness, training, or incentives to integrate research into their day-to-day professional activities.

The limitations of this study included a smaller sample population comprising only one University in the region. The data collected was quantitative in terms of agreement or disagreement of the participants, but it leaves many unanswered questions regarding the reasons for the choices made by these teachers. The study does not differentiate between the responses of the participants based on their demographic divisions. A response from a head of the institution and a Doctor of Medical Education is weighed equally with the response of a teacher in the first month of their career as an undergraduate teacher. A more homogenous sample with specific contexts and outcomes would be more likely to present targeted solutions. This study does not take into account the hierarchal structure of the organizations where the top-tier leadership is making the majority of the decisions and policies. It is generally accepted that a top-to-bottom approach gives more fruitful results in bringing about change in behaviors and practices. Qualitative research exploring the depth and complexity of decisions encountered by the faculty members can offer better insight.

## Conclusions

This study concludes that the respondent had positive attitude towards EBHPE. However, this positive attitude was not reflected in their practices. Healthcare professionals should be committed to educational excellence, and endeavour to rely on evidence-based literature regarding the planning and review of learning, teaching, and assessment strategies. This will help ensure a high standard of education that is justifiable and reliable.
